# Supplementary material for: Polycyclic Aromatic Hydrocarbons and Pancreatic Cancer: An Analysis of the Blood Biomarker, r-1,t-2,3,c-4-Tetrahydroxy-1,2,3,4-tetrahydrophenanthrene and Selected Metabolism Gene SNPs
Source: Nutrients. 2024 Feb 28;16(5):688. doi: 10.3390/nu16050688 (PMC10935191; doi:10.3390/nu16050688)
Supplement: Supplementary file 1 [file nutrients-16-00688-s001.zip › nutrients-2776613-supplementary.pdf]

Supplemental Table S1

Summary descriptives table by groups of `Pancreatic_Cancer'

	Control	Case	OR	p.overall
	N=198	***N=202***		
PheT	16.9 (11.1)	21.5 (25.1)	1.01 [1.00;1.03]	0.018
Smoked:				0.847
Never	101 (51.0%)	106 (52.5%)	Ref.	
Former	97 (49.0%)	96 (47.5%)	0.94 [0.64;1.40]	
Sex:				0.815
Female	82 (41.4%)	87 (43.1%)	Ref.	
Male	116 (58.6%)	115 (56.9%)	0.93 [0.63;1.39]	
Age	68.0 (10.7)	68.5 (10.4)	1.01 [0.99;1.02]	0.600
BMI	27.2 (4.67)	28.4 (5.66)	1.04 [1.00;1.09]	0.029
Pack_Years	10.6 (17.4)	9.65 (17.1)	1.00 [0.99;1.01]	0.581
Type_2_Diabetes:				<0.001
No	180 (90.9%)	144 (71.3%)	Ref.	
Yes	18 (9.09%)	58 (28.7%)	4.03 [2.27;7.14]	

Summary descriptives table by groups of `Pancreatic_Cancer'

	Control<br>*N=188*	Case<br>*N=93*	OR	p.overall
PheT per1000	10.3 (7.65)	15.2 (19.9)	1.03 [1.01;1.05]	0.023
Beer(drinks) per1000	0.66 (2.13)	0.55 (1.45)	0.97 [0.84;1.12]	0.586
Wine(drinks) per1000	0.72 (1.45)	0.43 (1.01)	0.82 [0.64;1.05]	0.063
Liquor(drinks) per1000	0.66 (1.42)	0.63 (1.28)	0.98 [0.82;1.18]	0.840
Vegetables(servings) per1000	1.10 (0.69)	1.25 (0.77)	1.31 [0.94;1.84]	0.124
Fruits(servings) per1000	0.97 (0.62)	1.21 (0.87)	1.59 [1.12;2.26]	0.016
oz_meat per1000	1.95 (0.82)	2.06 (0.89)	1.16 [0.87;1.56]	0.325
oz_meat_beefporklamb per1000	0.91 (0.55)	0.97 (0.55)	1.21 [0.78;1.89]	0.402
oz_meat_organs per1000	0.00 (0.01)	0.00 (0.01)	2.980000e+01 [0.000000e+00;4.254489e+18]	0.865
Luncheon/Franks(oz) per1000	0.34 (0.30)	0.41 (0.41)	1.67 [0.82;3.40]	0.193
oz_meat_poultry per1000	0.47 (0.40)	0.46 (0.43)	0.93 [0.50;1.71]	0.815
Seafood(oz) per1000	0.23 (0.19)	0.23 (0.16)	0.97 [0.24;3.86]	0.961
oz_meat_eggs per1000	0.26 (0.22)	0.34 (0.35)	2.75 [1.06;7.14]	0.062
oz_meat_soy per1000	0.01 (0.09)	0.03 (0.11)	10.1 [0.51;200]	0.111
Nuts(oz) per1000	0.31 (0.25)	0.23 (0.22)	0.23 [0.07;0.78]	0.013
Alcohol(drinks) per1000	0.48 (0.72)	0.38 (0.62)	0.79 [0.53;1.18]	0.220
svg_fruits_adjust per1000	0.90 (0.55)	0.98 (0.62)	1.28 [0.83;1.96]	0.279

	Control	Case	OR	p.overall
	N=188	*N=93*		
svg_veg_adjust per1000	0.53 (0.31)	0.50 (0.29)	0.73 [0.32;1.67]	0.445
Meiqx per1000	18.5 (19.7)	25.3 (46.1)	1.01 [1.00;1.02]	0.178
Phip per1000	39.7 (46.1)	62.1 (142)	1.00 [1.00;1.01]	0.143
Dimeiqx per1000	1.10 (1.23)	1.88 (5.34)	1.13 [0.96;1.32]	0.170
actual per1000	2617 (2576)	4575 (14330)	1.00 [1.00;1.00]	0.197
predict per1000	2609 (2500)	3620 (6722)	1.00 [1.00;1.00]	0.166
Bap per1000	12.7 (16.6)	15.8 (19.5)	1.01 [1.00;1.02]	0.194
Meiqx_red per1000	17.3 (19.5)	24.0 (45.6)	1.01 [1.00;1.02]	0.182
Phip_red per1000	22.2 (33.1)	39.2 (134)	1.00 [1.00;1.01]	0.236
Dimeiqx_red per1000	0.90 (1.16)	1.69 (5.26)	1.14 [0.96;1.36]	0.161
actual_red per1000	1918 (2322)	3663 (14069)	1.00 [1.00;1.00]	0.240
predict_red per1000	2227 (2446)	3199 (6520)	1.00 [1.00;1.00]	0.170
Bap_red per1000	10.5 (15.3)	12.5 (17.3)	1.01 [0.99;1.02]	0.348
Meiqx_white per1000	1.19 (1.75)	1.32 (2.21)	1.04 [0.91;1.18]	0.607
Phip_white per1000	17.5 (28.7)	23.0 (32.6)	1.01 [1.00;1.01]	0.171
Dimeiqx_white per1000	0.20 (0.47)	0.19 (0.34)	0.98 [0.55;1.77]	0.954
actual_white per1000	699 (1057)	912 (1341)	1.00 [1.00;1.00]	0.184
predict_white per1000	381 (575)	421 (533)	1.00 [1.00;1.00]	0.572

	Control	Case	OR	p.overall
	N=188	*N=93*		
Bap_white per1000	2.14 (4.63)	3.23 (6.87)	1.03 [0.99;1.08]	0.169
Meat(g) per1000	55.4 (23.3)	58.5 (25.3)	1.01 [1.00;1.02]	0.325
Red_Meat(g) per1000	35.7 (19.8)	39.1 (20.5)	1.01 [1.00;1.02]	0.182
White_Meat(g) per1000	19.7 (13.5)	19.3 (13.9)	1.00 [0.98;1.02]	0.826
g_red_proc per1000	9.83 (8.62)	11.6 (11.5)	1.02 [0.99;1.04]	0.193
g_red_raremed per1000	3.98 (6.20)	3.43 (6.40)	0.99 [0.95;1.03]	0.495
Very_Well_Done_Red_Meat(g) per1000	6.80 (7.09)	4.10 (4.09)	0.91 [0.87;0.97]	<0.001
BBQ_Red_Meat(g) per1000	4.30 (4.76)	3.20 (4.82)	0.95 [0.89;1.01]	0.070
Not_BBQ_Red_Meat(g) per1000	6.49 (6.90)	4.34 (4.44)	0.93 [0.88;0.98]	0.002
Br_Red_Meat(g) per1000	0.77 (1.84)	0.61 (1.63)	0.94 [0.81;1.10]	0.445
Not_Br_Red_Meat(g) per1000	10.0 (8.95)	6.93 (6.83)	0.95 [0.91;0.98]	0.002
Pf_Red_Meat(g) per1000	3.60 (5.48)	2.01 (2.91)	0.90 [0.82;0.98]	0.002
Not_Pf_Red_Meat(g) per1000	7.25 (6.59)	5.58 (6.21)	0.96 [0.91;1.00]	0.039
HEME_IRON per1000	264 (207)	297 (430)	1.00 [1.00;1.00]	0.474
RHEME_IRON per1000	244 (204)	275 (428)	1.00 [1.00;1.00]	0.511
WHEME_IRON per1000	19.3 (21.7)	22.2 (26.7)	1.01 [0.99;1.02]	0.364
NRITE_CLDCUT_POUL_MEAS per1000	0.01 (0.02)	0.00 (0.02)	0.00 [0.00;1666]	0.140
Nitrite_Ham_Lunch_Meats per1000	0.03 (0.06)	0.01 (0.03)	0.00 [0.00;0.01]	<0.001

	Control	Case	OR	p.overall
	N=188	*N=93*		
Nitrite_Coldcuts per1000	0.02 (0.03)	0.01 (0.02)	0.00 [0.00;0.00]	<0.001
Nitrite_Hotdogs per1000	0.00 (0.00)	0.00 (0.01)	1.837598e+25 [1.283000e+03;2.632432e+47]	0.044
Nitrite_Ham per1000	0.01 (0.01)	0.01 (0.03)	12038 [2.000000e-02;8147439049]	0.217
Nitrite_Bacon per1000	0.00 (0.01)	0.00 (0.00)	0.00 [0.00;364439686890]	0.292
Nitrite_Sausage per1000	0.00 (0.01)	0.00 (0.00)	0.00 [0.00;0.00]	0.002
Total_Nitrite per1000	0.08 (0.09)	0.05 (0.06)	0.00 [0.00;0.15]	0.001
Fruits/Vegetables(servings) per1000	2.07 (1.14)	2.46 (1.43)	1.28 [1.05;1.55]	0.022
